# The SPA17-AXL axis drives melanoma aggressiveness and SPA17-targeting vaccination exhibits antitumor efficacy

**DOI:** 10.3389/fimmu.2026.1914680

**Published:** 2026-09-09

**Authors:** Lan Zhang, Mengshu You, Bin Yan, Jian Zhang, Yi Yao, Qingyan Zhang, Zuozhong Xie, Shixiang Wang, Qian Gao

**Affiliations:** 1 Department of Dermatology, Xiangya Hospital, Central South University, Changsha, Hunan, China; 2 Department of Laboratory Medicine, Xiangya Hospital, Central South University, Changsha, Hunan, China; 3 Department of Dermatology, The First Affiliated Hospital of Guangzhou Medical University, Guangzhou Medical University, Guangzhou, Guangdong, China; 4 Department of Biomedical Informatics, School of Life Sciences, Central South University, Changsha, Hunan, China; 5 Department of Hematology, Xiangya Third Hospital, Central South University, Changsha, Hunan, China; 6 Department of Otolaryngology Head and Neck Surgery, The Second Xiangya Hospital of Central South University, Changsha, Hunan, China

**Keywords:** AXL, immune evasion, melanoma, SPA17, tumor vaccine

## Abstract

**Introduction:**

Sperm Autoantigenic Protein 17 (SPA17), a member of the cancer-testis antigen family, is frequently overexpressed in various malignancies, yet its biological functions, regulatory mechanisms, and clinical relevance in melanoma remain largely unexplored. This study aimed to investigate the expression pattern, prognostic value, and functional role of SPA17 in melanoma progression and immune modulation.

**Methods:**

We analyzed SPA17 expression in melanoma clinical specimens and correlated it with patient prognosis, metastatic burden, and immunotherapy response. Functional assays, including migration and proliferation tests, were performed in SPA17-knockdown melanoma cells *in vitro*, and tumor growth was evaluated in a subcutaneous syngeneic mouse model *in vivo*. Mechanistic studies involved co-culture systems with activated human lymphocytes and molecular interrogation of downstream effectors, with particular focus on AXL. The therapeutic potential of a SPA17-targeting peptide vaccine was also assessed in vivo, with tumor-infiltrating lymphocyte populations characterized by immunohistochemistry.

**Results:**

SPA17 was markedly upregulated in melanoma tissues, especially in metastatic lesions, and high expression correlated with poor prognosis, increased metastatic burden, and resistance to immunotherapy. Knockdown of SPA17 significantly suppressed melanoma cell migration in vitro and tumor growth *in vivo*, with no effect on proliferation. Mechanistically, SPA17 depletion enhanced intratumoral infiltration and cytotoxic activity of CD8^+^ T cells, and SPA17-knockdown melanoma cells showed reduced resistance to activated lymphocyte-mediated killing in co-culture. We identified AXL as a critical downstream effector, as SPA17 positively regulated AXL expression, and ectopic AXL overexpression rescued the migration defect and restored lymphocyte-mediated killing impaired by SPA17 knockdown. Therapeutically, administration of the SPA17-targeting peptide vaccine markedly restrained melanoma progression in vivo, accompanied by increased tumor-infiltrating CD8^+^ and GZMB^+^ CD8^+^ T cells.

**Discussion:**

Collectively, our findings suggest that SPA17 promotes melanoma progression and immune evasion through upregulation of AXL, and that SPA17-directed peptide vaccination elicits antitumor immune responses and inhibits tumor growth. These data support SPA17 as a candidate prognostic biomarker and a potential therapeutic target warranting further clinical investigation.

## 1 Introduction

Melanoma is the most aggressive malignant cutaneous tumor, often associated with a high tumor mutational burden (TMB), which endows it with significant immunogenicity. Multiple studies have shown that melanoma is generally considered an immunogenic tumor that can induce both humoral and cellular immune responses in the host (1, 2). In recent years, immune checkpoint inhibitors, including anti-PD-1/PD-L1 and anti-CTLA-4 antibodies, have significantly improved overall survival and long-term remission rates in patients with advanced melanoma, marking a milestone in immunotherapy for solid tumors (3, 4). However, although some patients achieve durable responses, the overall clinical response rate remains limited, and a considerable proportion of patients develop primary or acquired resistance (1, 5, 6).

Recent investigations into the mechanisms of immune resistance in melanoma have demonstrated that differences in the tumor antigen repertoire and defects in antigen processing and presentation are among the key factors contributing to the variability in responses to immunotherapy (7, 8). Specifically, when tumor cells impair their ability to present self-antigens, such as by downregulating MHC molecules or altering the expression of proteins involved in antigen processing, CD8^+^ T cells fail to effectively recognize them, thereby significantly diminishing the efficacy of immune checkpoint inhibitors (9, 10). These findings suggest that merely blocking T-cell inhibitory signals may be insufficient to eradicate tumors; enhancing the immune recognition of tumor antigens is also required. Consequently, exploring novel tumor antigens that can effectively stimulate specific T-cell responses has become an important direction for optimizing melanoma immunotherapy.

Cancer-Testis Antigens (CTAs) are protein antigens with normal expression restricted to adult testicular germ cells, and yet are aberrantly activated and expressed in a proportion of various types of human cancer (11, 12). Numerous studies have shown that CTAs can induce humoral and cellular immune responses, leading to their extensive investigation in tumor vaccines, T-cell receptor-engineered T-cell therapy (TCR-T), and combination therapy with immune checkpoint inhibitors (13–15). In terms of molecular characteristics, CTAs can be divided into two major categories: CT-X antigens (encoded on the X chromosome) and non-CT-X antigens (16). CT-X antigens exhibit widespread aberrant expression across diverse tumor types and strong immunogenicity, making them the most studied and applied type of CTAs to date (17, 18). For example, the MAGE-A3 vaccine has demonstrated good immunogenicity and safety in clinical trials for melanoma (19, 20). However, the subsequent Phase III clinical trial failed to achieve the expected clinical benefit regarding the primary endpoint, highlighting the gap between immune response and clinical efficacy, suggesting that the application of CTAs in immunotherapy still faces numerous challenges (21). In recent years, non-CT-X antigens, represented by PRAME, have become important candidate targets for the development of next-generation TCR-T and tumor vaccines due to their high expression in various solid tumors and their ability to induce specific T cell responses (22). Exploring more non-CT-X antigens with similar characteristics will help expand the repertoire of immunotherapy targets and advance the development of precision immunotherapy for tumors.

Sperm autoantigenic protein 17 (SPA17, also known as sperm surface protein 17 or Sp17) is a member of the non-CT-X family of cancer-testis antigens. Its expression is largely restricted to healthy male germ cells (specifically in sperm), with only trace levels detected in embryonic germ cells and ciliated epithelial cells under normal physiological conditions (23, 24). Indeed, accumulating evidence has demonstrated that SPA17 is aberrantly overexpressed in a range of neoplasms, including multiple myeloma, gynecological malignancies, and various solid tumors (1, 5–7, 24–27). Importantly, SPA17 possesses high immunogenicity and contains functional cytotoxic T lymphocyte (CTL) epitopes capable of eliciting specific T-cell responses (28–30). Moreover, SPA17-specific, HLA class I-restricted CTLs have been successfully generated from patients with diverse cancer types (29–31), underscoring its potential as a target for tumor vaccines and T-cell receptor-engineered T-cell (TCR-T) therapy. A pan-cancer analysis has indicated that SPA17 expression levels are significantly associated with survival outcomes and response rates to anti-PD-1 monoclonal antibody therapy in melanoma patients, suggesting its potential as both a prognostic biomarker and a predictor of immunotherapeutic efficacy (32). Nevertheless, the role of SPA17 in melanoma remains poorly understood, and the molecular function and specific immunoregulatory mechanisms of SPA17 in melanoma are required to elucidate the specific roles of SPA17 in melanoma progression and treatment resistance and to develop novel therapeutic strategies based on these insights.

AXL, a member of the TAM tyrosine kinase receptor family is aberrantly overexpressed in multiple cancers (33–36), where its upregulation is closely associated with tumor aggressiveness, metastatic potential, and resistance to various therapeutic modalities (37). In melanoma, high AXL expression is associated with the ability of tumor cells to transition from an epithelial phenotype to a more aggressive stromal phenotype, a transition closely linked to drug resistance events and promoting treatment resistance (38–40). AXL activation leads to downstream signaling through the PI3K/AKT, MAPK/ERK and STAT3 ­ pathways (41–43). However, there is currently limited research on the upstream regulators of AXL aberration expression (44, 45), which warrants further investigation.

To this end, we first examined the clinicopathological correlation and prognostic significance of SPA17 expression in melanoma tissues, thereby establishing its candidacy as a candidate prognostic factor. We further dissected the downstream signaling pathways and effectors that mediate SPA17-promoted malignancy. Finally, we evaluated the antitumor efficacy of a SPA17-based peptide vaccine in preclinical models to provide preliminary evidence for its translational potential.

## 2 Materials and methods

### 2.1 Cell culture

The human malignant melanoma cell lines (A375 and SK-MEL-28) were cultured in DMEM medium (Gibco, #C11995500BT) supplemented with 10% FBS (CellMax, SA101), 100 U of penicillin per ml and 100 mg/ml streptomycin (BI, #03-031-1B). Mouse melanoma cell line B16F10 was cultured in RPMI1640 medium (Gibco, #C11875500BT).

### 2.2 Mouse subcutaneous tumor model

C57BL/6 mice were purchased from the Shanghai SLAC and bred at the experimental animal center, Central South University. Mice used were female and specific-pathogen-free (SPF) grade, and were usually injected subcutaneously at 6–8 weeks old. A suspension of B16F10 cells was prepared in PBS at a density of 5×10^6^ cells/mL. Subsequently, 100 μL of this suspension was administered to each mouse via subcutaneous injection. The tumor was monitored regularly by measuring its length and width. Tumor volume (TV) was calculated by (L × W²)/2. In each treatment group, 4–5 mice were utilized to observe the tumor phenotype changes. The animal protocol was approved by the Ethics Committee of Xiangya Hospital (Central South University, Changsha, Hunan, China). All experiments strictly followed the guidelines for the investigation of experimental pain in conscious animals for minimizing animals’ suffering and improving animals’ welfare.

### 2.3 Vaccine preparation and immunization procedures

The SPA17-derived peptide (sequence: H-KEKEEVAAVKIQAAFRGHIAREEAKKMKTNSL-OH; #AL-195399), corresponding to human Sp17 amino acids 111–142, was commercially synthesized by ANLIANPEPTIDE CO., LTD using standard solid-phase Fmoc chemistry. The peptide sequence was selected based on our previous studies (11, 28). The peptide is predicted to be restricted to H-2K^b^/H-2D^b^, the MHC class I alleles of C57BL/6 mice, as determined by the SYFPEITHI and NetMHCpan algorithms. Sequence alignment between mouse (Mus musculus) and human (Homo sapiens) Sp17 in this region revealed approximately 74% homology when properly aligned (hSp17112–138 vs. mSp17109-136), with a conserved stretch of 12 identical amino acids. The peptide was purified by reversed-phase high-performance liquid chromatography (RP-HPLC) to a purity of >95%, as confirmed by analytical HPLC and mass spectrometry (MS), and was provided as a lyophilized powder stored at −20°C until use. ODN 2395 (sequence: 5′-TCGTCGTTTTCGGCGCGCGCCG-3′; #HY-150743C), used as a vaccine adjuvant, was purchased from MedChemExpress (Monmouth Junction, NJ, USA). The lyophilized ODN was reconstituted in endotoxin-free sterile water at a concentration of 10 µg/µL and stored at −20°C. On each immunization day, the SPA17 peptide was freshly dissolved in sterile phosphate-buffered saline (PBS, pH 7.4) at a concentration of 20 µg/µL and mixed with the ODN immediately prior to injection to achieve a final dose of 100 µg of SPA17 peptide and 30 µg of ODN per mouse in a total volume of 100 µL. The peptide and ODN were mixed in PBS immediately prior to injection. The formulated vaccine was used within 1 hour of preparation.

### 2.4 Clinical tissue samples

Paraffin sections of tumor tissue from melanoma patients treated with PD-1 mAb were collected from Xiangya Hospital. Stable disease (SD) with progression-free survival (PFS) longer than 6 months were classified as responders, while patients with SD with PFS shorter than 6 months and Progressive disease (PD) were categorized as non-responders. Detailed information on the melanoma samples is shown in **Supplementary Table 7** **(Supplementary Information).** All tissue samples were collected in compliance with the informed consent policy. Protocols were designed and performed according to the principles of the Declaration of Helsinki and were approved by the ethics review board of Xiangya Hospital.

### 2.5 Cell viability analysis

Cells were seeded into 96-well plates at a density of 5000 per well. Cells were incubated with 100 μL fresh medium with 10 μL CCK8 solution (#B34302, Selleckchem, USA) for 3 h at 37 °C after culturing 24 h, and then the optical density (OD) was measured at 450 nm by Model 680 Microplate Reader (Bio-Rad, USA).

### 2.6 Colony formation assay

Cells were seeded into 6-well plates at a density of 3,000–5,000 cells per well in an appropriate volume of suspension. Plates were gently rocked in a cross pattern to distribute cells evenly, then incubated under standard culture conditions for 2–3 weeks. Once macroscopic colonies became visible, the medium was removed, and the cells were washed twice with PBS. Colonies were fixed with 4% paraformaldehyde for 15 min, followed by two PBS washes after removal of the fixative. Cells were stained with crystal violet for 20 min, rinsed gently with PBS, and air-dried. The number of visible colonies was counted, and the colony formation efficiency was calculated.

### 2.7 Transwell migration assay

A volume of 100 μL cell suspension containing 5×10^4^ cells was added to the upper chamber of a Transwell insert. The lower chamber was filled with 500 μL medium supplemented with 30% FBS. The assembly was incubated at 37°C for 24–48 h. After incubation, non-migrated cells on the upper side of the membrane were removed by wiping gently with a cotton-tipped swab following two PBS washes. Cells on the lower surface were fixed with 4% paraformaldehyde for 15 min, washed twice with PBS, and then stained with crystal violet for 10–15 min. The staining solution was removed, and excess dye was rinsed by soaking in PBS. The insert was air-dried briefly, inverted onto a glass slide, and imaged using an upright microscope.

### 2.8 Plasmids and vectors

SPA17 shRNA (Human#1: CAUGCAUUCGAGGAGCAAGAA; Human#2: CAGUCACCAUCUUAGACUCUU; Mouse#1: GGUAGAGGACCGCUUCUAUTT) lentiviral vector carrying hairpins were purchased from GENEWIZ (Suzhou, China).

SPA17 siRNA (CAGUCACCAUCUUAGACUCUUTTAAGAGUCUAAGAUGGUGACUGTT) was purchased from GENESEED (Guangzhou, China). AXL-OE was purchased from GenePharma (Suzhou, China).

### 2.9 Cell transfections

For the stable transfection, cells at approximately 70-80% confluency were chosen for transduction. The culture medium was aspirated, and cells were washed twice with phosphate-buffered saline (PBS, BBI, #E607080). Based on the predetermined multiplicity of infection (MOI), the viral stock was diluted in serum-free complete medium to the desired concentration. Polybrene was added to a final concentration of 10 μg/mL and mixed gently. The diluted viral solution was applied to the cells, followed by incubation in a cell culture incubator for 24 h. After removal of the virus-containing medium, fresh serum-free complete medium was added. At 48 h postinfection, the medium was replaced with complete medium supplemented with 1 μg/mL puromycin (BasalMedia, #S250J0) to initiate selection. Transduction efficiency was assessed by RT-qPCR or Western blotting. For the transient transfection, cells with 70-80% confluence were transfected using Turbofect (Thermofisher) in opti-MEM medium (Gibco, #31985070) and the experimental procedure was carried out according to the protocol. Cells were collected or dealt after 24 h. Transduction efficiency was assessed by RT-qPCR or Western blotting.

### 2.10 T cell-mediated tumor cell killing assay

Peripheral blood (10 mL) was obtained from a healthy donor, and peripheral blood mononuclear cells (PBMCs) were isolated by means of lymphocyte separation medium (DAKEWE, #7111011). The isolated PBMCs were then suspended in T cell culture medium (STEMCELL, #10981) supplemented with 1000 U/mL IL-2 and a T cell activator, followed by culture in 6-well plates for at least 7 days to promote T cell expansion and activation. Prior to co-culture, cancer cells were plated and allowed to adhere overnight. These adherent cancer cells were subsequently incubated with the activated T cells for 48 h. In these experiments, we employed an effector-to-target ratio of 3:1(T cells to cancer cells). After co-culture, non-adherent T cells and cellular debris were removed by washing with PBS. Viable cancer cells were then fixed and stained with crystal violet, and their density was quantified by measuring the optical density at 570 nm using a spectrometer.

### 2.11 Flow cytometry analysis

For mouse samples, single cell suspensions of B16F10-xenograft tumors were obtained by rapid and gentle stripping, physical grinding and filter filtration. After blocking with CD16/CD32 (Biolegend, #101320) antibody and getting rid of dead cells with Zombie Aqua Fixable Viability Kit (Biolegend, #423102), cells were stained with APCCY7-CD45 (BD, #557659), APC-CD3 (Biolegend, #100236), BB700-CD4 (BD, #566407), PECY7-CD8(BD, #552877), PECY7-CD155(Biolegend, #131513), PE-CD173L(Biolegend, #107105), BB700-CD276 (Biolegend, #135616), PE/Dazzle594 -PD-L1(Biolegend, #124324) for 30 min at 4°C. After fixation and permeabilization by Foxp3/Transcription Factor Staining Buffer Set (eBioscience, #00-5523-00), intracellular GZMB was stained using PE/Dazzle594-GZMB (Biolegend, #372216), antibody. Stained cells were analyzed by BD FACSymphony A3 (BD Biosciences). Data were analyzed with FlowJo software (TreeStar, Ashland, OR).

### 2.12 Western blot analyses

Cells were lysed in RIPA Lysis Buffer (Beyotime, #P0013C) containing protease inhibitors (Biomake, #B14002) and phosphatase inhibitors (Biomake, #B15002). Subsequently, 5× SDS loading buffer was added to the protein solution and denatured by heating in a metal bath at 95 °C for 10 min. Protein samples were separated by 10% SDS-PAGE gel and transferred to Nitrocellulose membranes. After blocking with 5% skim milk and washing with TBST, the membranes were incubated with primary antibodies overnight at 4 °C and HRP-conjugated secondary antibodies (Santa Cruz Biotechnology) the next day, followed by signal detection with ECL solution in an Odyssey^®^ Fc system (LI-COR, USA). The primary antibodies used in the study were as follows: SPA17 antibody (Abcam, #ab172626), AXL antibody (CST, 8661T), GAPDH antibody (Proteintech, 60004-1-Ig).

### 2.13 Multi-color immunohistochemistry assay

Fix fresh tissue with formalin (#B0038, Powerful Biology, China) for 24 hours, then dehydrate, paraffin-embed and section to 5 μm tissue slides. Deparaffin (Xylene I 10min-Xylene II 10min-Absolute Ethanol I 5min-Absolute Ethanol II 5min), rehydration (95% Ethanol 5min-85%Ethanol 5min-75%Ethanal 5min-PBS 5min,3 times-PBST buffer 30s), and then heat-mediated antigen retrieval with Tris-EDTA buffer. Remove endogenous peroxidase activity and incubate with 5% goat serum for 30 min at room temperature. Incubate first primary antibody (CD8 Ab, 1:400, #AFRM0004, AiFang biological; GZMB Ab, 1:300, #AERM0352, AiFang biological; AXL Ab, 1:200, #AFRM0318, AiFang biological; SPA17 Ab, 1:50, #13367–1-AP, Proteintech) overnight at 4 °C in the dark, and then wash slides with PBST buffer three times on shaker for 5 min each time. Incubate secondary antibody (#AFIHC025, #0056, AiFang Biology, China) for 50 min at room temperature in the dark, and then wash slides with PBST buffer three times on a shaker for 5 min each time. Incubate corresponding fluorescence (#AFIHC025, #0056, AiFang Biology, China) for 3–10 min at room temperature in the dark, and then wash slides with PBST buffer three times on a shaker for 5 min each time. Incubate with multiple antibodies, skip to the antigen retrieval step and repeat next steps until staining all targets. The cell nucleus was marked with the nuclear dye 4′,6-diamidino-2-phenylindole (DAPI) (#AFIHC025, #0056, AiFang Biology, China) for 10 min at room temperature in the dark and wash slides three times with PBS buffer. Add anti-fluorescence quenching sealing medium (#AFIHC025, #0056, AiFang Biology, China) and cover with coverslip after the slices were slightly dried. The fluorescence was detected by the fluorescence digital slide scanner (#AF-KL-20-8, AiFang biological). The mean fluorescence intensity (MFI) was calculated from five representative fields per sample by using Image J, and then the data was normalized to the control group to calculate the relative fluorescence intensity.

### 2.14 Real-time RT–PCR analyses

The total RNA of mouse skin tissues and cells was extracted using TRIzol (Thermo Fisher Scientific, USA). 1 μg of RNA was reverse transcribed into cDNA using the Hifair^®^ ii 1st Strand cDNA Synthesis Kit (Yeasen, China). qPCR procedure was set up according to the instructions of 2X SYBR Green qPCR Master Mix (BiMake, China). And was performed on an Applied Biosystems^®^ QuantStudio^®^ 3 Real-Time PCR System (Thermo Fisher, USA). The delta-delta CT method was used to calculate the relative gene expression compared with *β-actin*. The PCR primers used in the study are shown in **Table 1**.

**Table 1:** Primer sequences for PCR amplification of target genes.

Primer name	Primer sequence (5’-3’)
H-*β-actin*_F	CACCATTGGCAATGAGCGGTTC
H-*β-actin*_R	AGGTCTTTGCGGATGTCCACGT
H-*SPA17*_F	GGAGTAAGGTAGAAGACCGCTTC
H-*SPA17*_R	TGGTGACTGATGTCTCTTCCTCC
H-*AXL*_F	GTTTGGAGCTGTGATGGAAGGC
H-*AXL*_R	CGCTTCACTCAGGAAATCCTCC

### 2.15 RNA sequencing and data processing

Total RNA was extracted from stable SPA17-knockdown and control melanoma cells (A375 and SK-MEL-28) and sent to Wuhan SeqHealth Tech Co., Ltd. (Wuhan, China) for library construction and sequencing. RNA libraries were constructed using the poly(A) enrichment method and sequenced on an MGISEQ-T7 platform to generate 150-bp paired-end reads. Data quality control and upstream processing were performed by the service provider. Raw data were filtered using fastp (version 0.23.0) (46) to remove adapters and low-quality reads. The clean reads were aligned to the human reference genome (GRCh38/hg38) using STAR (47). Gene abundance was quantified, and raw read counts were generated using featureCounts (48).

### 2.16 Transcriptomic profiling and functional characterization

Principal Component Analysis (PCA) was performed based on the raw count data to visualize sample clustering. Differential expression analysis was subsequently conducted using the DESeq2 R package (49). Genes satisfying the criteria of |log2 fold change (FC)| > 0.58, *p* value < 0.05, and adjusted *p* value < 0.2 were defined as differentially expressed genes (DEGs). Volcano plots were generated for each cell line, and common DEGs shared by both A375 and SK-MEL-28 cell lines were extracted.

Gene Set Enrichment Analysis (GSEA) was performed using the ClusterProfiler R package (50) against Gene Ontology (GO) terms (Biological Process, Molecular Function, and Cellular Component) based on pre-ranked Wald statistical metrics. Pathways exhibiting consistent regulation directions and statistical significance (adjusted *p<*0.05 in both cell lines) in both cell lines were deemed concordantly enriched. For multi-faceted visual presentation, the top 7 most statistically significant concordant pathways within each GO category are displayed.

### 2.17 Identification of candidate downstream mediators

To identify specific downstream mediators involved in immune evasion, the common downregulated DEGs were intersected with a curated “negative regulation of immune response” gene set. This gene set was constructed by integrating 531 unique genes from the ImmPort database (51) (https://www.immport.org), derived from 27 manually selected GO terms associated with negative immune regulation, tolerance, and homeostasis [listed in **Supplementary Table 1** (**Supplementary Information**)]. Venn diagrams were generated to visualize the intersection and identify candidate genes.

### 2.18 Mining of public databases for clinical association, prognosis, and immunotherapy response

Publicly available datasets were utilized to validate clinical correlations. The UCSCXenaShiny R package and web platform (52, 53) (https://shiny.hiplot.cn/ucsc-xena-shiny) were employed to download and analyze integrated TCGA and GTEx transcriptomic data, which were pre-processed via the UCSC Toil RNA-seq Recompute Pipeline to minimize computational batch effects (54). Specifically, differential expression analyses (e.g., tumor vs. normal) and pan-cancer molecular characterizations were performed. Standard Spearman correlation and partial Spearman correlation analyses (adjusted for tumor purity based on Consensus Purity Estimation) were calculated via this platform to evaluate gene co-expression in TCGA cohorts. Differential expression analyses between primary and metastatic tumors were conducted using the BEST database (55) (https://rookieutopia.hiplot.com.cn/app_direct/BEST/, accessed on January 2026), hosted on the Hiplot platform (56).

To perform patient-level survival analyses, independent melanoma datasets were manually curated. Specifically, TCGA-SKCM RNA expression profiles and baseline clinical data were downloaded from the GDC Hub via UCSC Xena (https://xena.ucsc.edu/), while the highly curated survival endpoints were obtained from the Pan-Cancer Atlas Hub. Furthermore, external melanoma validation cohorts, including three microarray datasets (GSE99898, GSE22153, and GSE46517) (57–59) and one RNA-seq dataset (GSE100797) (60), were downloaded from the Gene Expression Omnibus (GEO) database (https://www.ncbi.nlm.nih.gov/geo/).

To analyze the association between SPA17 expression and immunotherapy response, processed gene expression data of four independent melanoma cohorts (GSE93157 (61), GSE100797 (60), PRJEB23709 (62), and GSE78220 (63)) were obtained from the TIGER database (64) (http://tiger.canceromics.org/). These datasets comprise transcriptomic profiles of melanoma patients treated with different immunotherapeutic strategies: anti-PD-1 therapy for GSE93157, PRJEB23709, and GSE78220, and adoptive cell transfer (ACT) for GSE100797. Patients were classified into responders and non-responders based on clinical annotations, and differences in gene expression between groups were compared using the Wilcoxon rank-sum test.

### 2.19 Statistical analysis

Each experiment was performed at least three times. GraphPad Prism 9.0 (GraphPad Software, San Diego, CA, USA) and R version 4.5.1 were used to perform all the statistical analyses. All quantitative *in vitro* and *in vivo* data are presented as means ± standard deviation (SD). Prior to parametric testing, the Shapiro–Wilk test was applied to assess the normality of data distribution, and Levene’s test was used to evaluate the homogeneity of variances. For data meeting both assumptions, an unpaired Student’s t-test was used for comparisons between two independent groups, whereas one-way ANOVA followed by Tukey’s *post hoc* test was employed for comparisons among multiple groups. For longitudinal repeated-measures data, such as *in vivo* tumor volume and mouse body weight, a two-way repeated-measures ANOVA was performed, with Sidak’s *post hoc* test for multiple comparisons.

For transcriptomic and clinical data, differential gene expression was identified using the Wald test in DESeq2. Associations between gene expression and clinical features (stratified by median expression) were assessed with the Wilcoxon rank-sum test. Correlations between gene expression levels were evaluated using Spearman’s rank correlation analysis or partial Spearman’s rank correlation analysis when adjusting for tumor purity. Survival outcomes, stratified by both optimal and predefined median cutoff values, were evaluated using Kaplan-Meier curves, and differences were compared with the log-rank test. Univariate and multivariable Cox proportional hazards regression models were utilized to calculate hazard ratios (HR) and 95% confidence intervals (CI), assessing the independent prognostic value of target genes. A p-value of less than 0.05 was considered statistically significant (with notations: *p < 0.05, **p < 0.01, ***p < 0.001, ****p < 0.0001, ns: not significant).

## 3 Results

### 3.1 SPA17 is highly expressed in melanoma, which is associated with melanoma metastasis, immunotherapy response, and prognosis

**Figure 1:**
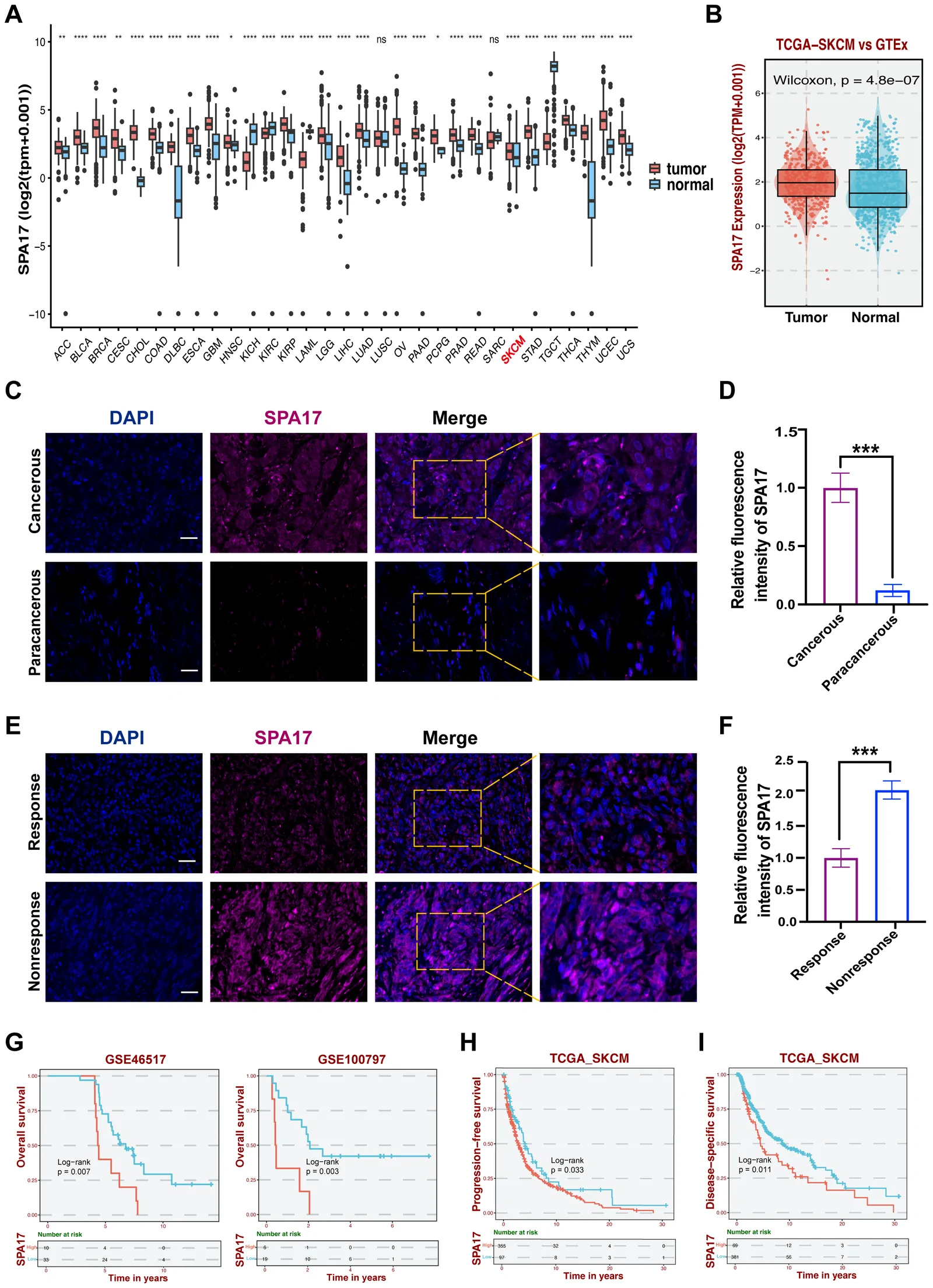
SPA17 is highly expressed in melanoma and significantly associated with prognosis. **(A)** Pan-cancer SPA17 expression in tumor (red) and normal (blue) tissues derived from the combined TCGA and GTEx datasets. **(B)** SPA17 expression in skin cutaneous melanoma (red) and normal skin (blue) tissues derived from the combined TCGA and GTEx datasets. Statistical significance was determined by the Wilcoxon rank-sum test for **(A, B)** (*p < 0.05, **p < 0.01, ****p < 0.0001, ns: not significant). **(C, D)** Representative immunofluorescence images and quantitative analysis of SPA17 in human melanoma tissues. Scale bar, 50 μm. The values presented are the means ± SD (n=4). Unpaired t test, ***p < 0.001. **(E, F)** Representative immunofluorescence image and quantitative analysis of SPA17 immunofluorescence staining of SPA17 in human melanoma tissues after immunotherapy treatment. Scale bar, 50 μm. The values presented are the means ± SD (n=4). Unpaired t test, ***p < 0.001. **(G)** Kaplan-Meier curves for overall survival in melanoma patients from the GSE100797 and GSE46517 datasets. **(H)** Kaplan-Meier curve for progress-free survival in the TCGA-SKCM cohort. **(I)** Kaplan-Meier curve for disease-specific survival in the TCGA-SKCM cohort. Patients were stratified by high (red) and low (blue) SPA17 expression. For survival analyses **(G–I)**, *p*-values were calculated using the log-rank test.

Given the limited research on SPA17 in tumors, we first analyzed the expression differences of SPA17 between tumor and normal tissues across 33 TCGA cohorts using the UCSCXenaShiny platform. The results showed that SPA17 was significantly highly expressed in various tumor tissues, including skin cutaneous melanoma (SKCM), suggesting that SPA17 may play a potential role in the development and progression of these tumors (**Figures 1A, B**). Subsequently, we detected the expression level of SPA17 in human melanoma samples via multiplex Immunohistochemistry(mIHC). Consistent with previous findings, SPA17 was significantly highly expressed in the cancerous area compared to the paracancerous area (**Figures 1C, D**). To further characterize its molecular landscape, we conducted a comprehensive cross-omics analysis across these cohorts (**Supplementary Figure 1A**). Notably, SPA17 exhibited a high frequency of copy number deletion in SKCM (54.2%), ranking third among all cancer types analyzed. This suggests a complex regulatory mechanism associated with its aberrant expression.

We next explored the relationship between SPA17 expression and response to immunotherapy by analyzing transcriptomic data from four independent melanoma cohorts (GSE93157, GSE100797, PRJEB23709, and GSE78220) treated with anti-PD-1 blockade or ACT [**Supplementary Table 2** (**Supplementary Information**)]. Notably, SPA17 expression was significantly higher in non-responders compared to responders in the GSE93157 cohort (*p* < 0.05) (**Supplementary Figure 1B**). Although the differences lacked statistical significance in the other three datasets, a consistent trend of elevated SPA17 levels in non-responders was still observed (**Supplementary Figures 1C–E**). Validation at the protein level further demonstrated that SPA17 expression was significantly upregulated in melanoma tissues obtained from patients who did not respond to immunotherapy, compared to those who exhibited a clinical response (**Figures 1E, F**). Next analysis revealed a pronounced elevation in SPA17 expression within tumor metastatic lesions compared to primary tumor sites, suggesting its potential involvement in melanoma progression and metastatic dissemination (**Supplementary Figures 1F, G**). Subsequently, we evaluated the clinical relevance of SPA17 expression in melanoma prognosis. Kaplan-Meier survival analysis based on optimal cutoff values demonstrated that high SPA17 expression was significantly correlated with poor overall survival (OS), progression-free survival (PFS), and disease-specific survival (DSS) in melanoma patients (**Figures 1G, H**, **Supplementary Table 3** [**Supplementary Information**)]. Further evaluation using pre-defined median cutoffs or continuous-variable Cox regression models attenuated the statistical significance, yet a consistent trend of poorer prognosis (Hazard Ratios > 1) was maintained [**Supplementary Tables 3, 4** (**Supplementary Information**)]. These findings suggest that SPA17 may represent a candidate prognostic factor and is associated with adverse clinical outcomes in melanoma.

### 3.2 Knockdown of SPA17 inhibits melanoma cell migration and tumor growth

**Figure 2:**
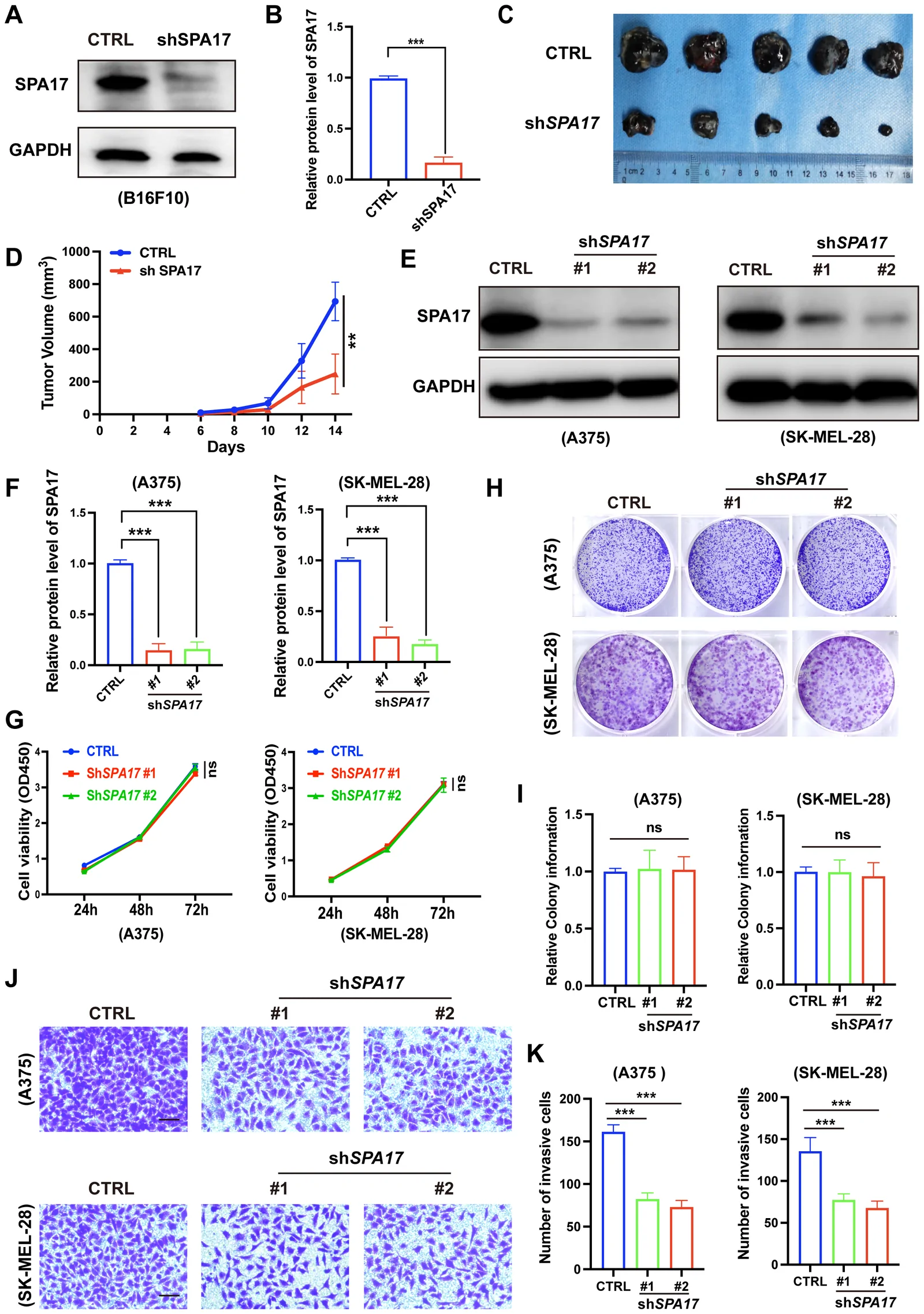
SPA17 knockdown inhibits melanoma cell migration and melanoma growth. **(A, B)** Western Blot analysis of SPA17 expression in the B16F10 melanoma cell line transfected with shSPA17 or control (CTRL) shRNA. Data are shown as means ± SEM; n = 3 independent experiments. Unpaired t test, ***p < 0.001. **(C)** Photographs of representative tumors (n=5). **(D)** Summary of volume data of B16F10 tumors harvested after euthanizing the mice. The values presented are the means ± SD (n=5). Two-way ANOVA, **p < 0.01. **(E, F)** Western blot analysis of SPA17 expression in A375 and SK-MEL-28 melanoma cell lines transfected with shSPA17 compared to control (CTRL) shRNA. Data are shown as means ± SEM; n = 3 independent experiments. One-way ANOVA, ***p < 0.001. **(G)** CCK8 assay. The values presented are the means ± SD (n=3). Two-way ANOVA, ns: not significant. **(H, I)** The representative clone formation images and relative clone formation ability were quantified. The values presented are the means ± SD (n=3). One-way ANOVA, ns: not significant. **(J)** Images of the Transwell migration assay for the A375 and SK-MEL-28 cells. Scale bar, 50 μm. **(K)** Quantified results of the Transwell migration assay. The values presented are the means ± SD (n=3). One-way ANOVA, ***p < 0.001.

To further investigate the function of SPA17 in melanoma, we constructed SPA17 knockdown B16 melanoma cell line and these cells were inoculated subcutaneously into C57BL/6J mice to establish a subcutaneous tumor model (**Figures 2A, B**). Our results showed that the knockdown of SPA17 expression on B16 cells limited tumorigenic ability as the tumor growth was significantly inhibited. These results indicate that SPA17 expression is one of the factors that promote tumor growth (**Figures 2C, D**). Next, we constructed SPA17 knockdown A375 and SK-MEL-28 cell lines to further investigate the effects of SPA17 on the biological behavior of melanoma cells *in vitro* (**Figures 2E, F**). We found that SPA17 downregulation did not affect the ability of cell proliferation ability and colony formation (**Figures 2G–I**). Interestingly, the migration ability was significantly reduced while SPA17 expression was deregulated (**Figures 2J, K**). The results above indicate that SPA17 can enhance the migration of melanoma cells *in vitro* and promote melanoma growth *in vivo.*

### 3.3 Knockdown of SPA17 is associated with increased CD8^+^ T-cell infiltration and enhanced cytotoxic activity in melanoma

**Figure 3:**
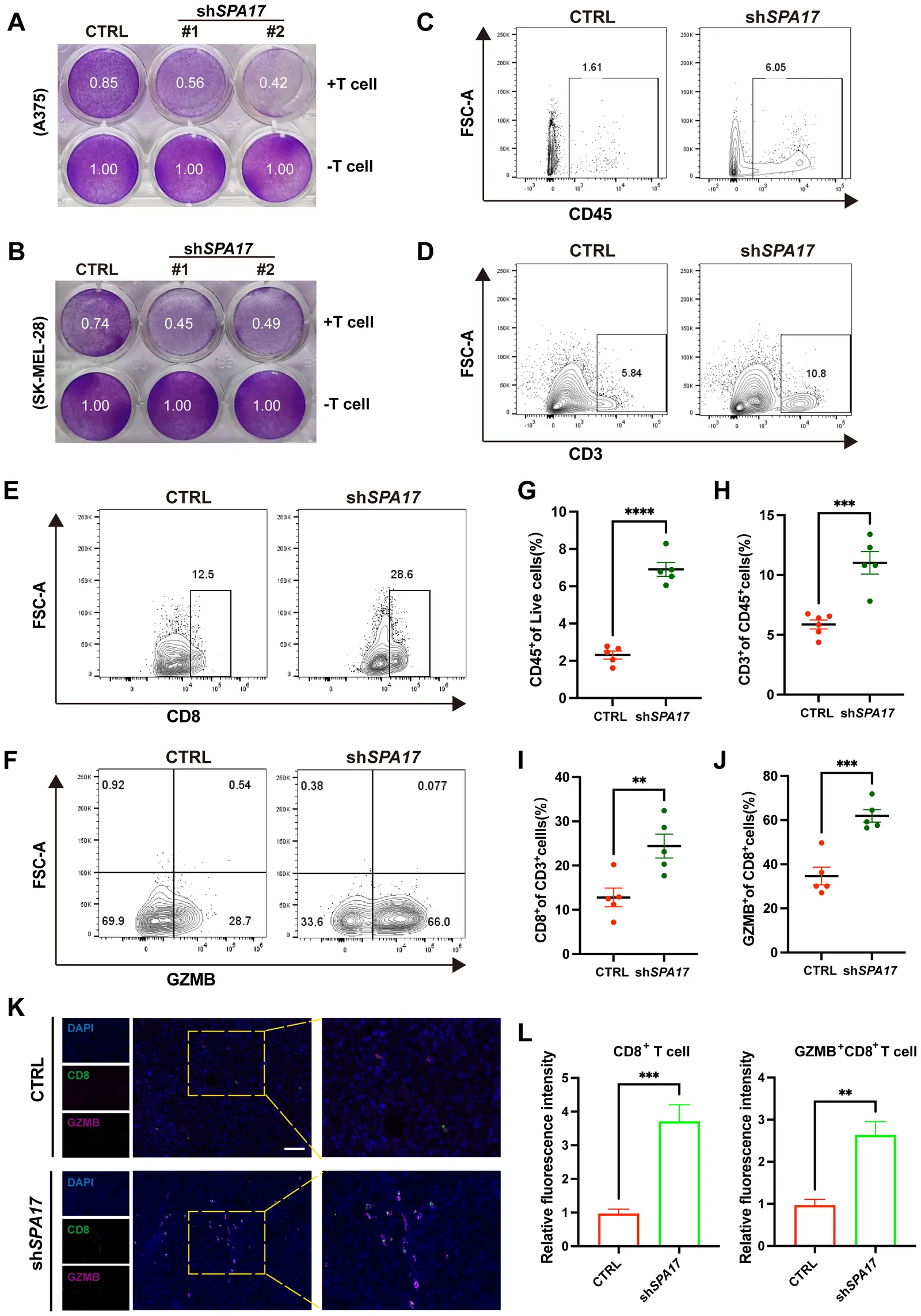
SPA17 knockdown enhances T cell-mediated antitumor immunity and promotes immune cell infiltration in melanoma. **(A, B)** T cell-mediated cancer cell-killing assay results (n=3). A375 or SK-MEL-28 melanoma cells cocultured with activated T cells for 48 h were subjected to crystal violet staining. The ratio of A375 or SK-MEL-28 to T cells is 1:3. **(C, G)** FACS analysis of CD45^+^ cells in live cells from the B16F10 syngeneic mouse melanoma model. The values presented are the means ± SD (n=5). Unpaired t test, ****p < 0.0001. **(D, H)** FACS analysis of CD3^+^ T cells in CD45^+^ cells from the B16F10 syngeneic mouse melanoma model. The values presented are the means ± SD (n=5). Unpaired t test, ***p < 0.001. **(E, I)** FACS analysis of CD8^+^ T cells in CD3^+^ T cells from the B16F10 syngeneic mouse melanoma model. The values presented are the means ± SD (n=5). Unpaired t test, **p < 0.01. **(F, J)** FACS analysis of GZMB^+^CD8^+^ T cells in CD8^+^ T cells from the B16F10 syngeneic mouse melanoma model. The values presented are the means ± SD (n=5). Unpaired t test, ***p < 0.001. **(K, L)** Representative immunofluorescence images and quantitative analysis of melanoma tissues from control (CTRL) and shSPA17 groups. Scale bar, 50 μm. The values presented are the means ± SD (n=5). Unpaired t test, **p < 0.01, ***p < 0.001.

Given the potential association between SPA17 expression and clinical immunotherapy response (**Supplementary Figures 2B–E**), we sought to further investigate its impact on tumor immunity. In coculture experiments, we observed that downregulation of SPA17 in melanoma cells reduced their resistance to human lymphocytes (peripheral blood mononuclear cells) in T cell-mediated tumor cell killing assays (**Figures 3A, B**). We acknowledge that this assay utilized bulk PBMCs from a single healthy donor following non-specific T cell activation, and thus does not establish SPA17-specific T cell immunity. Nonetheless, these results indicate that SPA17 expression in melanoma cells influences their susceptibility to lymphocyte-mediated cytotoxicity *in vitro*. Next, in subcutaneous tumorigenesis experiments, we found that tumors with SPA17 knockdown had increased infiltration of CD45^+^ cells and CD3^+^ T cells (**Figures 3C, D, G, H**). Moreover, our results revealed that tumors with SPA17 knockdown contained a greater abundance of CD8^+^ T cells (**Figures 3E, I**), as well as a higher proportion of CD8^+^ T cells expressing GZMB (**Figures 3F, J**), which is the cytotoxic effector molecule of CD8^+^ T cells. Consistently, we used mIHC to detect the abundance of CD8 and GZMB in mouse tumor tissues and found that tumor tissues from mice with SPA17 knockdown had more CD8+ T cells and GZMB^+^CD8^+^ T cells infiltration (**Figures 3K, L**). Notably, downregulation of SPA17 in B16 cells did not affect the expression of common immune checkpoints (PD-L1, CD155, CD137L, CD276) (**Supplementary Figures 2A–H**). In summary, our findings indicate that SPA17 knockdown is associated with enhanced CD8^+^ T-cell infiltration in melanoma.

### 3.4 SPA17 upregulates AXL expression in melanoma

**Figure 4:**
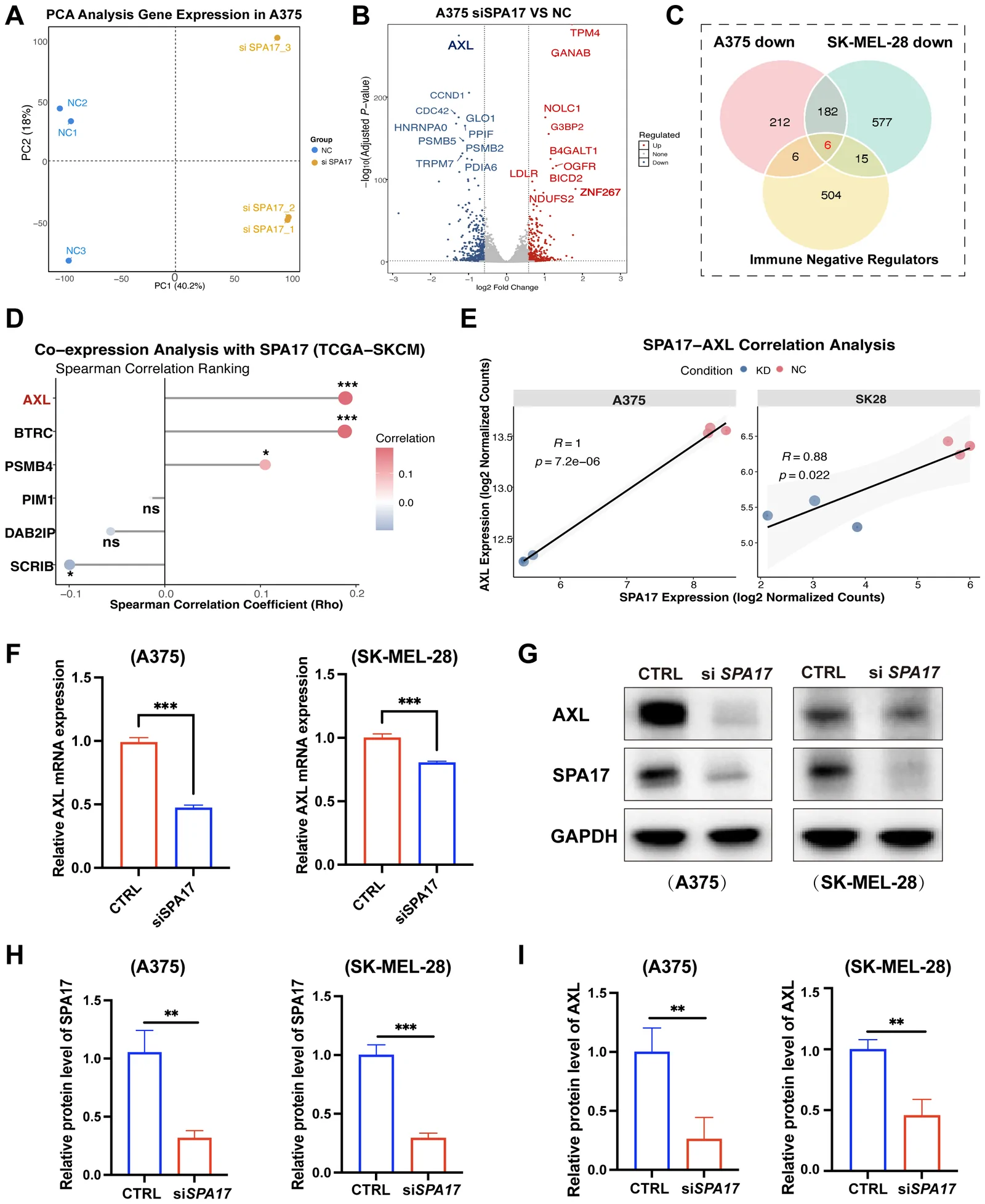
SPA17 expression positively correlates with AXL in melanoma. **(A)** Principal component analysis (PCA) of A375 cells transfected with siSPA17 or NC (n=3). **(B)** Volcano plot of differentially expressed genes in A375 cells (siSPA17 vs. NC). The top 10 genes ranked by adjusted p-value are labeled. **(C)** Venn diagram illustrating the intersection of downregulated genes in SPA17-knockdown A375 and SK-MEL-28 cells with a curated set of immune negative regulators. The central overlap identifies 6 shared candidate genes. **(D)** Spearman correlation analysis of SPA17 with the 6 candidate genes in the TCGA-SKCM dataset. Genes are ranked by correlation coefficient, with AXL showing the strongest positive correlation. (*p < 0.05, ***p < 0.001, ns: not significant). **(E)** Correlation analysis of SPA17 and AXL expression in A375 and SK-MEL-28 cells. Pearson correlation coefficients (R) and *p* values are shown. **(F)** The expression of AXL by qRT-PCR. Data are shown as means ± SEM; n = 3 independent experiments. Unpaired t test, ***p < 0.001. **(G)** Western blot analysis of SPA17 and AXL expression in A375 and SK-MEL-28 melanoma cells transfected with siSPA17 compared to control (CTRL) siRNA. **(H, I)** Densitometric quantification of the blotting. Data are shown as means ± SEM; n = 3 independent experiments. Unpaired t test, **p < 0.01, ***p < 0.001.

**Figure 5:**
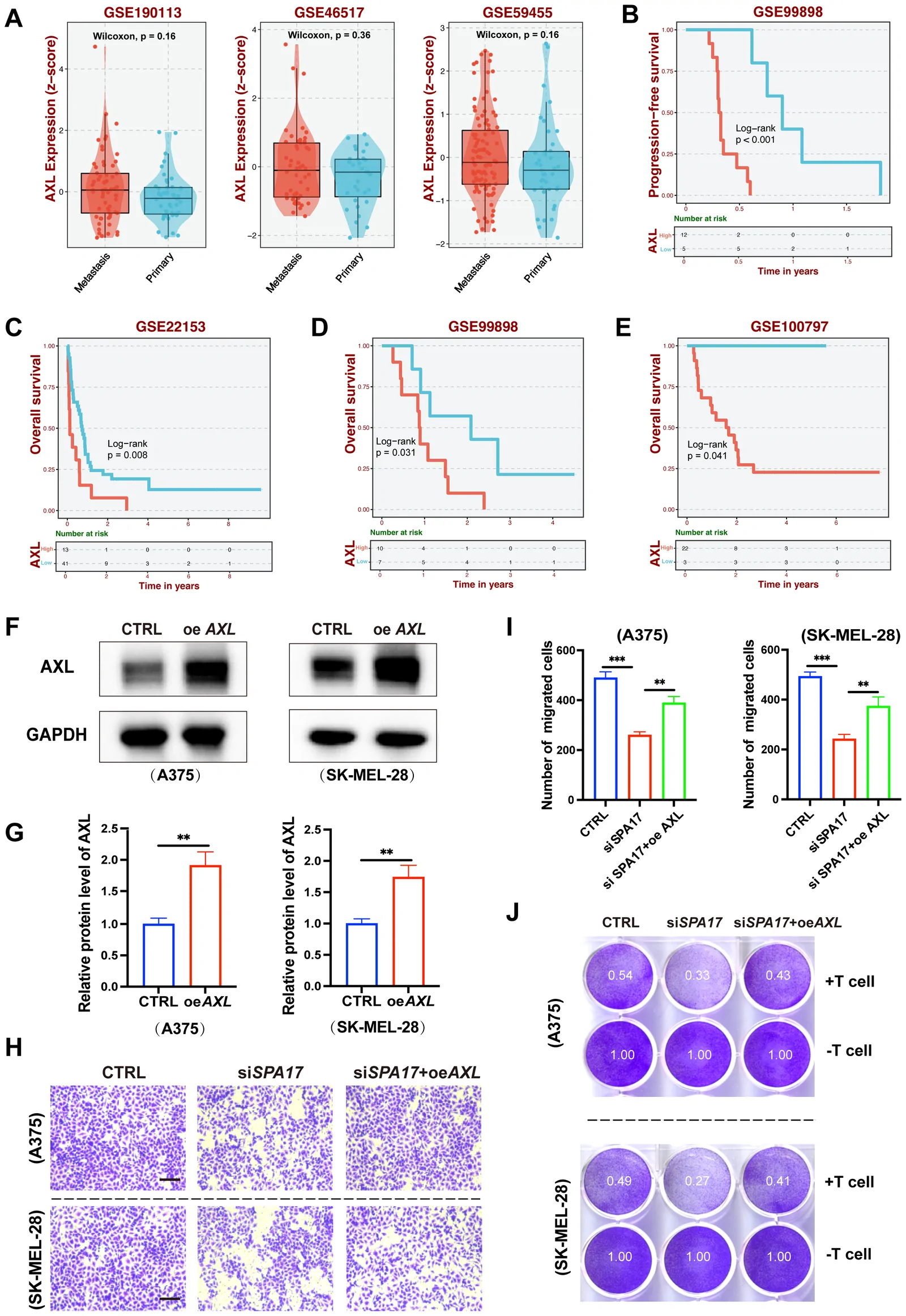
AXL is associated with melanoma progression and rescues SPA17 knockdown effects. **(A)** Analysis of AXL expression differences between metastatic and primary melanoma tissues in the GSE190113 (left), GSE46517 (middle), and GSE59455 (right) datasets. Statistical significance was determined by the Wilcoxon rank-sum test. **(B)** Kaplan-Meier curve for progression-free survival in the GSE99898 cohort. **(C–E)** Kaplan-Meier curves for overall survival in melanoma patients from the GSE22153 **(C)**, GSE99898 **(D)**, and GSE100797 **(E)** datasets. Patients were stratified by high (red) and low (blue) AXL expression. p-values were calculated using the log-rank test for survival analyses. **(F, G)** Western blot analysis of AXL expression following its overexpression in A375 and SK-MEL-28 cells. Data are shown as means ± SEM; n = 3 independent experiments. Unpaired t test, **p < 0.01. **(H, I)** Images of the Transwell assay for the A375 and SK-MEL-28 cells. Scale bar, 100 μm. The values presented are the means ± SD (n=3). One-way ANOVA, **p < 0.01, ***p < 0.001. **(J)** T cell-mediated cancer cell-killing assay results (n=3). A375 or SK-MEL-28 melanoma cells cocultured with activated T cells for 48 h were subjected to crystal violet staining. The ratio of A375 or SK-MEL-28 to T cells is 1:3.

Furthermore, we performed RNA-seq analysis on cells with SPA17 knockdown to delineate the molecular mechanisms driven by SPA17 (**Supplementary Figure 3A**). Principal component analysis revealed distinct transcriptomic profiles between the knockdown and control groups in both A375 and SK-MEL-28 cell lines (**Figure 4A**; **Supplementary Figure 3B**). Differential expression analysis was then performed for each cell line, and intersection analysis revealed 89 common upregulated and 188 common downregulated genes shared by both datasets [**Figure 4B**; **Supplementary Figure 2C**; **Supplementary Table 5** (**Supplementary Information**)]. Given the enhanced T cell-mediated cytotoxicity observed in our functional assays, we specifically sought to identify the molecular drivers responsible for the SPA17-mediated immune resistance. To this end, we intersected the 188 common downregulated DEGs with the “negative regulation of immune response” gene set. This analysis identified six candidate genes: AXL, BTRC, PSMB4, PIM1, DAB2IP, and SCRIB (**Figure 4C**). Notably, among these candidates, AXL was the most significantly downregulated in both cell lines (A375: log2FC = -1.26, adjusted *p* = 2.18E-275; SK-MEL-28: log2FC = -0.96, adjusted *p* = 1.16E-04), showing the most pronounced downregulation in the A375 cell line [**Figure 4B**; **Supplementary Table 5** (**Supplementary Information**)]. We next prioritized the most clinically relevant target among these candidates by examining their co-expression with SPA17 in the TCGA-SKCM cohort. Distinct from other candidates that showed weak or insignificant correlations (e.g., PIM1, DAB2IP), AXL exhibited a robust and significant positive correlation with SPA17 in melanoma patients (**Figure 4D**). This consistent correlation was further validated in our melanoma cell lines (**Figure 4E**). Importantly, this positive correlation is not restricted to melanoma but is conserved across diverse cancer types. In this pan-cancer context, SKCM consistently ranked among the top 8 cancer types (**Supplementary Figures 4A, B**). Next, we examined the AXL mRNA level in melanoma cells which knockdown SPA17, and we found that there is a significant downregulation of AXL in the condition of SPA17 knockdown (**Figure 4F**). Then we examined AXL protein levels after SPA17 knockdown in A375 and SK28 cell lines, which also showed a significant decrease (**Figures 5G–I**). Moreover, GSEA analysis indicated that downregulated pathways in both cell lines were significantly enriched in biological processes related to cell cycle phase transition, DNA replication, and chromosome segregation, as well as cellular components such as the spindle and chromosomal regions (**Supplementary Figure 3D**). Conversely, upregulated pathways (**Supplementary Figure 3E**) were consistently concentrated in proton motive force-driven ATP synthesis, ATP biosynthetic processes, and respiratory chain complexes, suggesting altered cellular metabolic states following SPA17 silencing.

### 3.5 AXL mediates SPA17-associated effects on melanoma cell migration and T-cell-mediated cytotoxicity

Given the positive correlation between SPA17 and AXL expression observed in melanoma cells and patient tissues (**Figures 4D, E**), we next investigated whether AXL functions as a downstream mediator of SPA17-associated phenotypes. Analysis of public melanoma datasets revealed that AXL expression was elevated in metastatic lesions compared to primary tumors (**Figure 5A**) and tended to correlate with patient prognosis, although this association varied across different statistical cutoffs and models [**Figures 5B–E**; **Supplementary Tables 3, 4** (**Supplementary information**)]. Functionally, AXL overexpression rescued the migratory defect caused by SPA17 knockdown (**Figures 5F–I**) and also reversed the increased susceptibility of SPA17-knockdown melanoma cells to lymphocyte-mediated killing (**Figure 5J**). Collectively, these rescue experiments support a functional link between SPA17 and AXL in modulating melanoma cell migration and sensitivity to T-cell-mediated cytotoxicity, highlighting the SPA17-AXL axis as a potential contributor to melanoma aggressiveness.

### 3.6 SPA17 peptide vaccine exerts antitumor activity and is accompanied by increased CD8^+^ T cell infiltration

**Figure 6:**
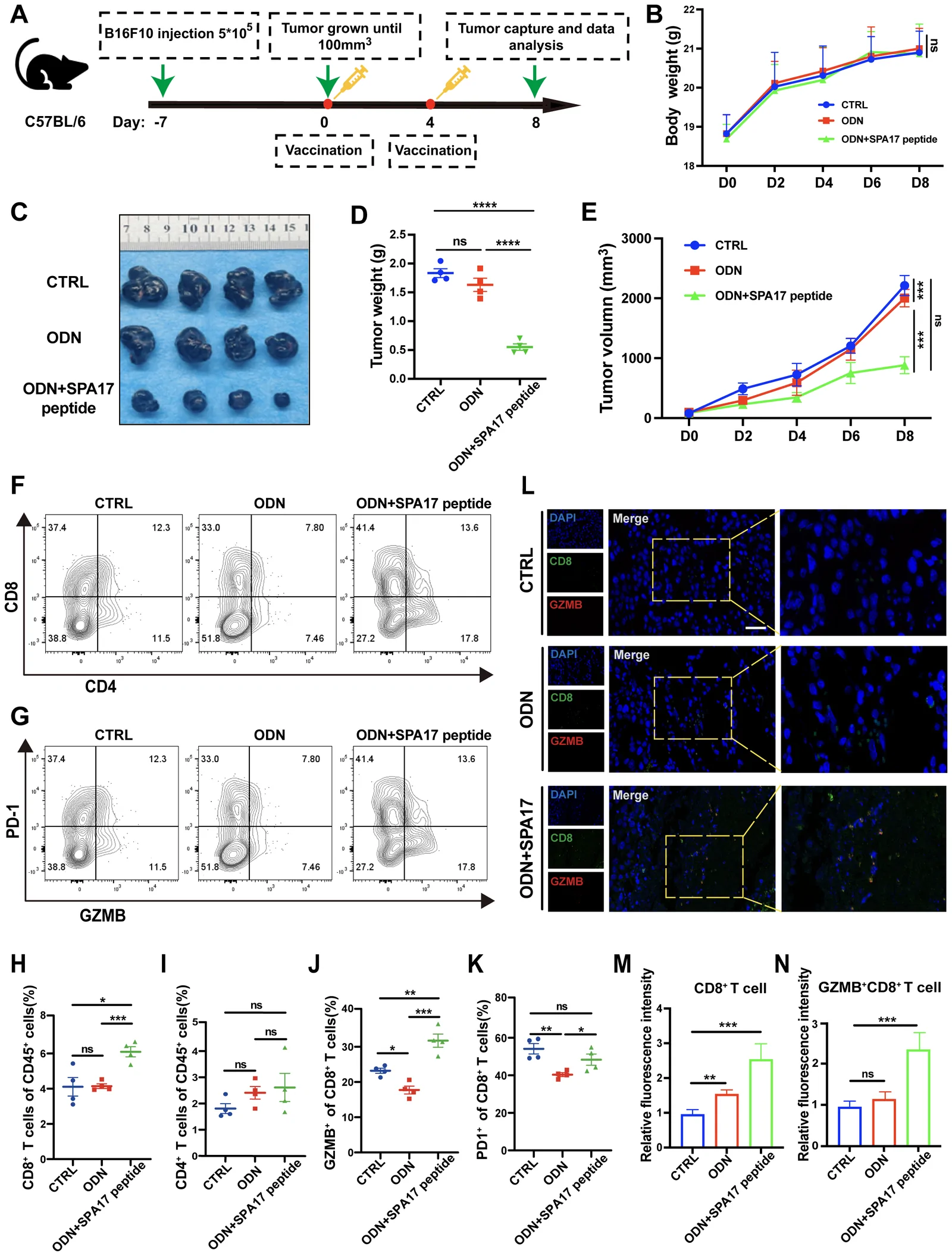
SPA17 peptide vaccination suppresses tumor growth and enhances CD8^+^ T-cell infiltration. **(A)** C57BL/6 mice were implanted with 5×10^5^ B16F10 cells and respectively vaccinated with the SPA17 peptide (n=4). **(B)** Mice’s body weight was measured every 2 days. The values presented are the means ± SD (n=4). Two-way ANOVA, ns: not significant. **(C)** Photographs of representative tumors. **(D)** Tumor weights were measured in the B16F10 tumor burden mouse model. The values presented are the means ± SD (n=4). One-way ANOVA, ****p < 0.0001, ns: not significant. **(E)** Summary of volume data of B16F10 tumors harvested after euthanizing the mice. The values presented are the means ± SD (n=4). Two-way ANOVA, ***p < 0.001, ns: not significant. **(F, H, I)** FACS analysis of CD8^+^ or CD4^+^ T cells in CD45^+^ cells from the B16F10 syngeneic mouse melanoma model. The values presented are the means ± SD (n=4). One-way ANOVA, *p < 0.05, ***p < 0.001, ns: not significant. **(G, J, K)** FACS analysis of GZMB^+^ or PD-1^+^ cells in CD8^+^ T cells from the B16F10 syngeneic mouse melanoma model. The values presented are the means ± SD (n=4). One-way ANOVA, *p < 0.05, **p < 0.01, ***p < 0.001, ns: not significant. **(L, M, N)** Representative immunofluorescence images and quantitative analysis of melanoma tissues from mice treated with SPA17 peptide vaccine versus CTRL (n=4). The relative MFIs presented are the means ± SD (n=4). One-way ANOVA, **p < 0.01, ***p < 0.001, ns: not significant.

Based on our preliminary research, we designed a peptide vaccine targeting SPA17 antigenic epitopes and evaluated its short-term antitumor activity in a B16F10 syngeneic mouse melanoma model (**Figure 6A**). Over the course of the experiment, mice treated with the SPA17 peptide vaccine showed no significant difference in body weight compared to the control (CTRL) and ODN groups (**Figure 6B**), indicating an absence of obvious acute systemic toxicity. We observed significantly slower tumor growth, as well as reduced final tumor volume and weight in mice treated with SPA17 peptide vaccine compared with mice in CTRL and ODN groups (**Figures 6C–E**). Since immune-mediated tumor clearance mainly depends on activated CD8^+^ T cells, which exert cytotoxic effects by secreting granzyme B (GZMB), we investigated the number and activity of CD8^+^ T cells infiltrating tumor regions in mice treated with the SPA17 peptide vaccine. Flow cytometry analysis revealed that the proportion of CD8^+^ T cells, GZMB^+^ CD8^+^ T cells and PD-1^+^ CD8^+^ T cells in tumors was increased, while the proportion of CD4^+^ T cells in the tumors of mice treated with the SPA17 peptide vaccine did not change significantly (**Figures 6F–K**). This result was corroborated by mIHC analysis, which revealed an increased proportion of CD8^+^ T cells and GZMB^+^ CD8^+^ T cells within the tumor tissues of vaccine-treated mice (**Figures 6L–N**). Notably, the abundance of LAG-3^+^ CD8^+^ T cells did not differ significantly between groups (**Supplementary Figures 5A, B**). Furthermore, the expression levels of CD155, CD137L, CD276 and PD-L1 on melanoma cells did not differ significantly between ODN and vaccine groups (**Supplementary Figures 5C–H**). Collectively, these results indicate that the SPA17 peptide vaccine exhibits short-term antitumor activity in this subcutaneous melanoma model, with a concomitant increase in tumor-infiltrating CD8^+^ T cells.

## 4 Discussion

**Figure 7:**
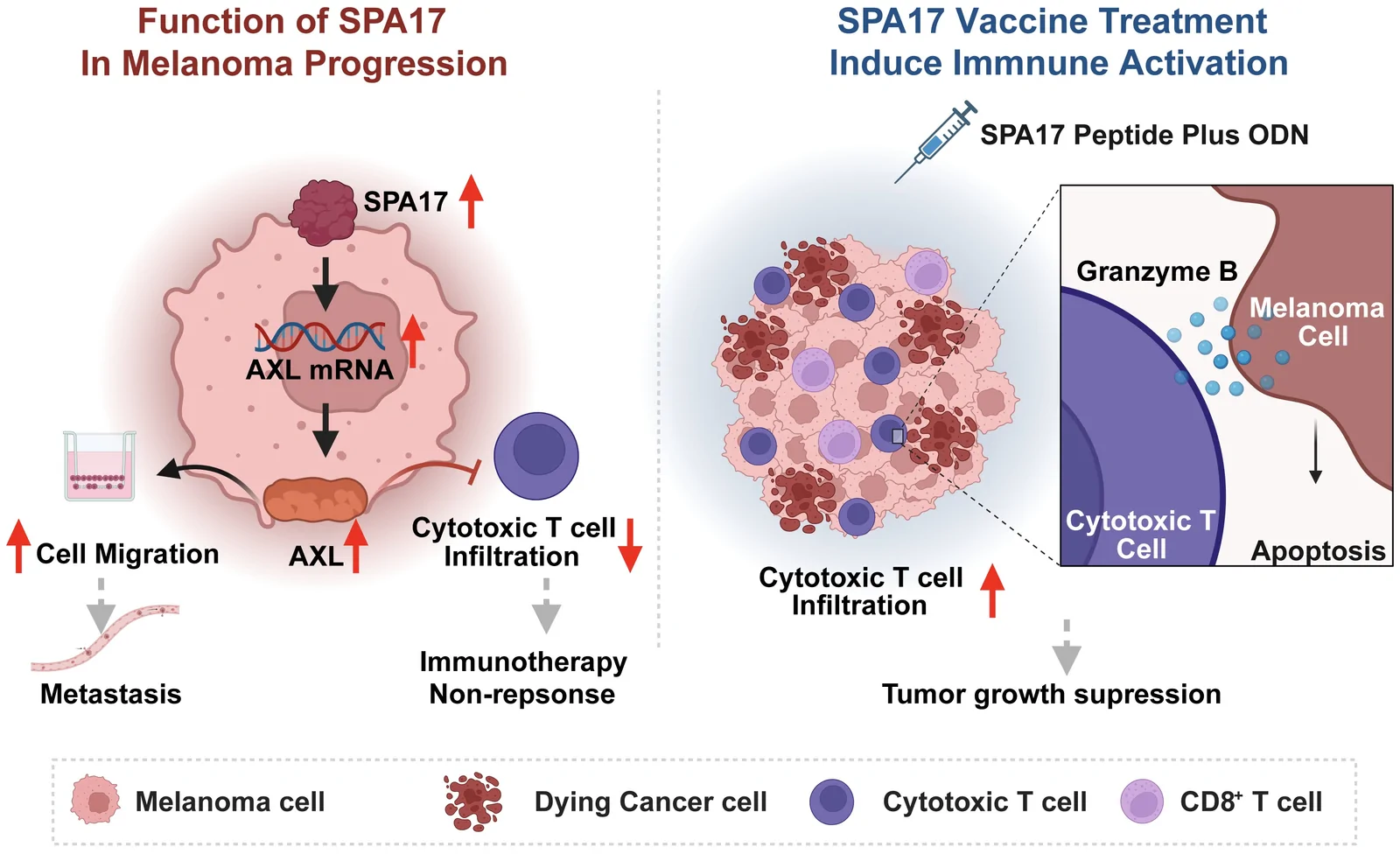
Proposed working model of the SPA17 promotes melanoma progression and SPA17 Peptide Vaccine Therapy.

Our study demonstrates that SPA17 is highly expressed in multiple cancers, is associated with AXL expression, enhances tumor cell migration, and correlates with reduced cytotoxic function of intratumoral CD8^+^ T cells. Furthermore, administration of a SPA17-targeting peptide vaccine was associated with preclinical antitumor activity in a subcutaneous melanoma model, accompanied by increased infiltration of cytotoxic CD8^+^ T cells. Overall, these findings suggest that SPA17 may contribute to melanoma progression and warrant further exploration as a potential therapeutic target (illustrated in **Figure 7**).

As a CTA, SPA17 has garnered widespread attention for its role in tumors owing to its tissue-specific expression and strong immunogenicity. Previous studies have noted aberrant expression of SPA17 in multiple cancers (23, 32, 65, 66). Our research found that SPA17 is significantly overexpressed in melanoma tissues and metastatic lesions, and its elevated expression is associated with potential prognostic value regarding adverse clinical outcomes (including OS, PFS, and DSS). This suggests that SPA17 may serve as a potential diagnostic and candidate prognostic factor and could represent a risk factor associated with disease progression (67–73). Immunosensors targeting SPA17 have already been developed and may be used for clinical diagnostic applications in melanoma (74–76). Notably, we observed higher SPA17 expression in tumor tissues from patients unresponsive to immunotherapy, linking its expression level to resistance to immunotherapy in melanoma. This suggests a potential involvement in immune evasion process and highlights its possible utility as a potential candidate factor for immunotherapy response (32). Additionally, *in vitro* functional experiments showed that SPA17 does not affect cellular proliferation but specifically enhances migratory capacity, corroborating its high expression in metastatic lesions, consistent with a previous study (77). Collectively, these findings suggest that SPA17 may play an important role in melanoma progression.

At the mechanistic level, we linked the function of SPA17 to a key tumor-associated molecule, AXL, which is a member of the TAM (TYRO3, AXL, and MERTK) family of receptor tyrosine kinases and has emerged as a promising target in cancer research (78). Previous research indicates that AXL is involved in tumor growth, metastasis, epithelial-mesenchymal transition, and immunosuppression, playing a significant role in cancer development, progression, drug resistance, and treatment tolerance (78–80). Mechanistically, in prostate cancer, AXL regulates cell proliferation through the Akt/NF-κB pathway (81). In hepatocellular carcinoma (HCC), AXL promotes metastasis by facilitating angiogenesis via activation of the PI3K/Akt/SOX2/DKK-1 signaling pathway (82). In head and neck cancer, AXL confers resistance to cetuximab by upregulating the HER3 ligand neuregulin (83). In agreement with prior studies, AXL activation facilitates melanoma migration. Our findings suggest that SPA17 upregulates the expression of AXL, and that this may cooperatively promote melanoma cell migration (84) and suppressing anti-tumor immunity (85). Moreover, a study has indicated that the AXL signaling pathway can regulate the infiltration of CD8^+^ T cells in the TME (85). Our findings demonstrate that downregulation of SPA17 leads to a reduction in AXL expression while simultaneously increasing the infiltration of cytotoxic CD8^+^ T cells in the TME. Of note, in melanoma patients with poor response to BRAF inhibitors, there is often a molecular characteristic of AXL overexpression, which may be related to the upregulation of SPA17, meaning that a therapy strategy targeting both SPA17 and AXL may potentially improve outcomes for these patients (39). Notably, the selective AXL inhibitor bemcentinib (BGB324/R428) recently received Fast Track designation from the U.S. FDA for use in combination with PD-1/PD-L1 inhibitors, underscoring its growing importance in cancer research.

Contrary to previous studies, we found no significant change in PD-L1 expression on SP17-expressing tumor cells, suggesting that the role of SP17 may be heterogeneous across different tumor types (24). Overall, our findings support the concept of a “SPA17-AXL” functional axis that may enhance tumor cell migration and suppresses anti-tumor immunity, and the exploration of combination immunotherapy based on this axis represents an emerging direction in melanoma therapeutics.

Numerous studies have demonstrated that CTAs can elicit both humoral and cellular immune responses, and have been extensively investigated in fields such as tumor vaccines, TCR-T, as well as combination therapies with immune checkpoint inhibitors (14, 16). Previous studies have demonstrated that anti-Sp17 monoclonal antibodies exhibit certain antibody-dependent cellular cytotoxicity (ADCC) and complement-dependent cytotoxicity against Sp17-expressing ovarian cancer cells (25). Multiple laboratories have successfully generated cytotoxic T cells targeting SPA17, although their efficacy across various tumor types warrants further exploration (29–31, 86). This Strong immunogenicity of SPA17 allows tumor cells to be recognized and killed by specific CTLs, making it a candidate for the development of tumor vaccines targeting SPA17. Similar to previous studies, our results showed that the SPA17 peptide vaccine has promising antitumor activity in a subcutaneous melanoma model, associated with inhibited tumor growth (26). A marked increase in the abundance of CD8^+^ T cells, particularly GZMB^+^ cytotoxic CD8^+^ T cells, was observed in the tumors following SPA17 peptide vaccine treatment (87). These observations support the concept that targeting SPA17 may remodel the tumor immune microenvironment and enhance antitumor immune responses, potentially offering a novel vaccine-based strategy. However, we acknowledge that further mechanistic studies are needed to establish causality and to develop optimized combination strategies.

Our study has several limitations. First, we found that high expression of SPA17 is associated with poor prognosis and resistance to immunotherapy; however, the molecular background and immune microenvironment vary significantly across different melanoma subtypes. Therefore, future validation of its prognostic and predictive value in multi-center, large-scale clinical cohorts encompassing various subtypes is necessary. Second, the specific mechanisms through which the SPA17-AXL axis modulates melanoma cell migration and CD8^+^ T-cell function warrant further exploration. Third, although our study demonstrates the antitumor activity of the SPA17 peptide vaccine in a subcutaneous melanoma model, its antitumor effects in models that more closely mimic clinical scenarios—such as genetically engineered spontaneous models, orthotopic transplantation, or metastatic models—deserve further investigation. Fourth, we recognize that our *in vitro* T-cell killing assay has several inherent limitations, including the use of bulk PBMCs from a single donor, non-antigen-specific T-cell activation, and the potential for alloreactive responses due to HLA mismatches. Therefore, this experiment does not establish SPA17-specific T-cell immune evasion. Rather, it demonstrates that SPA17 modulates the sensitivity of melanoma cells to activated lymphocytes, a finding that is corroborated by our *in vivo* observations of enhanced CD8^+^ T-cell infiltration and GZMB^+^ CD8^+^ T-cell activity in SPA17-knockdown tumors. Future studies using SPA17-specific CTL lines or TCR-transgenic models will be required to formally test whether SPA17 mediates antigen-specific immune evasion. Fifth, we found no significant change in PD-L1, CD155, CD137L, or CD276 expression on SPA17-expressing tumor cells. However, as we examined only a limited set of immune checkpoint molecules, this finding does not rule out the involvement of other checkpoint pathways. Future studies with broader checkpoint panels are warranted to fully characterize the immunomodulatory mechanisms of SPA17. Sixth, we acknowledge several limitations in our vaccine study. We did not include a control group receiving an irrelevant or scrambled peptide together with ODN, which would have more rigorously controlled for adjuvant-induced non-specific immune activation. We also did not include a peptide-only group, which would have allowed assessment of the adjuvant’s contribution to the observed antitumor effects. Additionally, we did not test the vaccine in SPA17-knockdown tumors; such an experiment would be necessary to formally demonstrate that the vaccine’s efficacy is strictly dependent on SPA17 expression in the tumor. In the absence of these controls, we cannot definitively attribute the antitumor effects solely to SPA17-specific immunity, as bystander T-cell activation, epitope spreading, or adjuvant-driven innate immune responses may also contribute. Furthermore, we did not formally demonstrate that the vaccine induces SPA17-specific T-cell responses. Future studies using peptide-restimulated ELISpot, MHC-tetramer staining, or intracellular cytokine staining will be required to establish antigen specificity. We therefore interpret our findings as demonstrating that SPA17-directed peptide vaccination is associated with antitumor activity and enhanced CD8^+^ T-cell infiltration, rather than providing formal proof of antigen-specific mechanism. Finally, we acknowledge that our vaccine study was designed as a short-term experiment. As such, our findings demonstrate short-term antitumor activity in a subcutaneous melanoma model, but they do not address long-term safety, autoimmune responses, reproductive toxicity, injury to normal SPA17-expressing tissues immunological memory, survival benefit, tumor recurrence, or protection against tumor rechallenge. These important questions will require future studies with extended observation periods, dedicated experimental designs, and comprehensive safety assessments before any clinical translation can be considered.

In summary, our study suggests a significant role for the SPA17 in melanoma progression and highlights its potential as a candidate prognostic factor for melanoma. Furthermore, we provide preliminary evidence for the antitumor activity of an SPA17 peptide vaccine in a murine melanoma model. Notably, further preclinical and translational studies are warranted before clinical development can be considered.

## Data Availability

The datasets presented in this study can be found in online repositories. The names of the repository/repositories and accession number(s) can be found in the article/**Supplementary Material**.
