# Supplementary material for: The SPA17-AXL axis drives melanoma aggressiveness and SPA17-targeting vaccination exhibits antitumor efficacy
Source: Front Immunol. 2026 Sep 9;17:1914680. doi: 10.3389/fimmu.2026.1914680 (PMC13597369; doi:10.3389/fimmu.2026.1914680)
Supplement: Supplementary file 1 [file DataSheet1.zip › Supplementary Information/Supplementary Figures and Figure legends.docx]

**Supplementary Figures and Figure Legends**

**Figure S1 The tumor mutational burden of SPA17 and its correlation with melanoma metastasis and the efficacy of immunotherapy. (A)** The cross-omics landscape of SPA17 across TCGA cancer types. The plot displays differential mRNA expression (Tumor vs. Normal), mutation status, CNVs, transcript isoforms, and DNA methylation levels. **(B-E)** Comparison of SPA17 expression levels between immunotherapy responders and non-responders in four independent melanoma cohorts: **(B)** GSE93157, **(C)** GSE100797, **(D)** PRJEB23709, and **(E)** GSE78220. **(F, G)** Analysis of SPA17 expression differences between metastatic and primary melanoma tissues in the GSE190113 **(F)**, GSE46517 **(G)** datasets. Statistical significance was determined by the Wilcoxon rank-sum test or Student's t-test as indicated.

**Figure S2 Impact of SPA17 loss on immune checkpoints in mouse melanoma.** **(A)** FACS of PD-L1^+^ cells in tumor cells from B16F10 xenografts and **(B)** quantification. The values presented are the means ± SD (n=5). Unpaired t test, ns: not significant. **(C)** FACS of CD155^+^ cells in tumor cells from B16F10 xenografts and **(D)** quantification. The values presented are the means ± SD (n=5). Unpaired t test, ns: not significant. **(E)** FACS of CD137L^+^ cells in tumor cells from B16F10 xenografts and **(F)** quantification. The values presented are the means ± SD (n=5). Unpaired t test, ns: not significant. **(G)** FACS of CD276^+^ cells in tumor cells from B16F10 xenografts and **(H)** quantification. The values presented are the means ± SD (n=5). Unpaired t test, ns: not significant.

**Figure S3 RNA-seq analysis on cells with SPA17 knockdown. (A)** The expression of SPA17 by qRT-PCR. The values presented are the means ± SD (n=3). One-way ANOVA, ****p* < 0.001. **(B)** Principal component analysis (PCA) of SK-MEL-28 cells transfected with siSPA17 or NC (n=3). **(C)** Volcano plot of differentially expressed genes in SK-MEL-28 cells. The top 10 genes ranked by adjusted p value are labeled. **(D, E)** Concordant GSEA pathways of common downregulated **(D)** and upregulated **(E)** Gene Ontology (GO) terms shared by A375 and SK-MEL-28 cells. Pathways with consistent regulation directions and statistical significance (adjusted *p*<0.05 in both cell lines) were selected. The top 7 enrichment terms for Biological Process (BP), Cellular Component (CC), and Molecular Function (MF) categories are displayed.

**Figure S4 Pan-cancer analysis of AXL. (A, B)** Pan-cancer analysis of the correlation between SPA17 and AXL expression. The lollipop charts display the partial Spearman correlation adjusted for tumor purity **(A)** and the standard Spearman correlation **(B)** across TCGA cancer types. In both plots, dot size corresponds to the statistical significance (-log10 adjusted p value), and color indicates the direction of correlation (red: positive; blue: negative). The SKCM cohort is highlighted (red box).

**Figure S5 Impact of SPA17 peptide vaccine on immune checkpoints in mouse melanoma.** **(A)** FACS of LAG3^+^ cells in CD8^+^ T cells from B16F10 xenografts and **(B)** quantification. The values presented are the means ± SD (n=4). One-way ANOVA, ns: not significant. **(C)** FACS of CD276^+^ cells in tumor cells from B16F10 xenografts and **(D)** quantification. The values presented are the means ± SD (n=4). One-way ANOVA, **p* < 0.05, ns: not significant. **(E)** FACS of PDL1^+^ cells in tumor cells from B16F10 xenografts and **(F)** quantification. The values presented are the means ± SD (n=4). One-way ANOVA, ns: not significant. **(G)** FACS of CD155^+^ cells in tumor cells from B16F10 xenografts and **(H)** quantification. The values presented are the means ± SD (n=4). One-way ANOVA, ns: not significant.
